# *In Silico* Identification of New Putative Pathogenic Variants in the *Neu1* Sialidase Gene Affecting Enzyme Function and Subcellular Localization

**DOI:** 10.1371/journal.pone.0104229

**Published:** 2014-08-25

**Authors:** Dario Bonardi, Viola Ravasio, Giuseppe Borsani, Alessandra d'Azzo, Roberto Bresciani, Eugenio Monti, Edoardo Giacopuzzi

**Affiliations:** 1 Department of Molecular and Translation Medicine, Unit of Biotechnology, University of Brescia, Brescia, Lombardy, Italy; 2 Department of Molecular and Translation Medicine, Unit of Biology and Genetics, University of Brescia, Brescia, Lombardy, Italy; 3 Department of Genetics, St Jude Children's Research Hospital, Memphis, Tennessee, United States of America; University Hospital S. Maria della Misericordia, Udine, Italy

## Abstract

The *NEU1* gene is the first identified member of the human sialidases, glycohydrolitic enzymes that remove the terminal sialic acid from oligosaccharide chains. Mutations in *NEU1* gene are causative of sialidosis (MIM 256550), a severe lysosomal storage disorder showing autosomal recessive mode of inheritance. Sialidosis has been classified into two subtypes: sialidosis type I, a normomorphic, late-onset form, and sialidosis type II, a more severe neonatal or early-onset form. A total of 50 causative mutations are reported in HGMD database, most of which are missense variants. To further characterize the *NEU1* gene and identify new functionally relevant protein isoforms, we decided to study its genetic variability in the human population using the data generated by two large sequencing projects: the 1000 Genomes Project (1000G) and the NHLBI GO Exome Sequencing Project (ESP). Together these two datasets comprise a cohort of 7595 sequenced individuals, making it possible to identify rare variants and dissect population specific ones. By integrating this approach with biochemical and cellular studies, we were able to identify new rare missense and frameshift alleles in *NEU1* gene. Among the 9 candidate variants tested, only two resulted in significantly lower levels of sialidase activity (p<0.05), namely c.650T>C and c.700G>A. These two mutations give rise to the amino acid substitutions p.V217A and p.D234N, respectively. NEU1 variants including either of these two amino acid changes have 44% and 25% residual sialidase activity when compared to the wild-type enzyme, reduced protein levels and altered subcellular localization. Thus they may represent new, putative pathological mutations resulting in sialidosis type I. The *in silico* approach used in this study has enabled the identification of previously unknown *NEU1* functional alleles that are widespread in the population and could be tested in future functional studies.

## Introduction

The *NEU1* gene (MIM 608272) is the first identified member of the human sialidases [1]. Sialidases (EC 3.2.1.18) are a family of glycohydrolitic enzymes that remove the terminal sialic acid from oligosaccharide chains of various natural substrates. In human four sialidase enzymes (NEU1–4) have been identified so far, each protein with a distinct substrate specificity and subcellular localization: the lysosomal NEU1, the cytosolic NEU2, the membrane-bound NEU3 and NEU4 [2]. All of them share the β-propeller structure organized in six blades, each composed of four antiparallel β-sheets, typical of sialidases [3]. A recent study reconstructing the evolution of the sialidase protein family in Metazoa confirmed the high conservation of this structure and the key features of sialidase active site [4]. Essential catalytic residues are strictly conserved and comprise: three Arg, that bind the carboxylate group common to all sialic acids, a Tyr/Glu nucleophile pair and an Asp that acts as the acid/base catalyst [2]. Mutations in *NEU1* gene have been identified in patients affected by neuraminidase deficiency or sialidosis (MIM 256550), a severe lysosomal storage disorder showing autosomal recessive heritability. Defective NEU1 enzymatic activity in the lysosomes causes accumulation of sialylated compounds, such as gangliosides and glycoproteins that results in severe cytotoxicity and cell death [5], [6]. Sialidosis affects approx. 1/4,200,000 individuals and is classified in two subtypes [7]. Sialidosis type I is a milder, late-onset, normosomatic form of the disorder, characterized by visual defects, myoclonus syndrome, cherry-red oculo-macular spots, ataxia, hyperreflexia, and seizures. Sialidosis type II is the severe early-onset form, associated with dysostosis multiplex, Hurler-like phenotype, mental retardation, and hepatosplenomegaly. The age of onset and severity of clinical manifestations correlate with the sialidase residual activity, with type II sialidosis usually presenting a completely inactive enzyme [2], [8], [9]. The association of NEU1 with PPCA, a protein encoded by the *CTSA* gene, is essential for the correct trafficking to lysosomes, where the sialidase enzyme is processed to its active form [10]. Thus, NEU1 variants resulting in a defective interaction with PPCA also lead to disease, even if the essential residues for the catalytic activity are not affected [5], [11], [12]. To date, 50 causative mutations are reported in HGMD [13] database, most of which are missense variants, suggesting a high allelic heterogeneity. Recent studies have also defined a role for NEU1 in various multifactorial diseases, such as atherosclerosis [14], obesity [15], diabetes [16], [17] and Alzheimer's disease [18], as well as in other important cellular processes, such as cancer and immunological response [19]. To further characterize the *NEU1* gene and identify functionally relevant protein isoforms, we decided to study its genetic variability in the human population. This approach is today made feasible by the huge amount of genomic data freely available to the scientific community, generated by large sequencing projects. The 1000 Genomes Project (1000G) [20] provides high-coverage exome sequences and middle or low-coverage genome sequences from about 1000 healthy individuals, while the NHLBI GO Exome Sequencing Project (ESP) [21] provides high-coverage exome sequences from about 6500 individuals, including both healthy controls and subjects affected by heart, lung and blood disorders. These studies rely on next generation sequencing (NGS) technologies to generate the complete sequence of genome/exome, allowing the identification of single nucleotide polymorphisms (SNPs), small insertion/deletions (indels) and large genomic rearrangements (CNVs). The samples analyzed are collected from individuals of different ethnicity allowing the estimation of allele frequencies in the overall population as well as in the single ancestry groups. Overall, the two datasets result in a cohort of 7595 individuals with detailed genomic data, making possible to identify rare variants, as well as to dissect population specific ones. Integrating this approach with biochemical and cellular studies led us to the identification in *NEU1* gene of new rare alleles carrying missense and frameshift mutations responsible for impaired enzyme activity, thus representing new putative causative mutations responsible for sialidosis.

## Materials and Methods

### Retrieving of NEU1 variants from 1000G and ESP6500 databases

Single nucleotide variants (SNV) for *NEU1* human gene (*NEU1*, NM_000434.3) were recovered directly from the data available from the 1000G and ESP6500 public repository, updated at March 2013. The genomic coordinates of the gene were used to extract the variants of interest from the VCF (variant call format) files containing global SNVs and indels annotations. Variants in *NEU1* gene were annotated using wANNOVAR [22] to obtain complete functional information. In house developed tools were used to manipulate the VCF files and analyze data on genotype and allele frequency for the global population and for the 4 subpopulations described in the 1000G dataset (African, AFR; American, AMR; European, EUR; Asiatic, ASN).

### Analysis of variants and identification of new candidate mutations in *NEU1*

Variants identified in the *NEU1* exons were categorized in UTR, synonymous, missense, stop-lost, splice-site, stop-gained and frameshift based on functional annotations from wANNOVAR [22]. The latter 3 groups are subsequently referred to as Loss of Function (LoF) variants. The functional impact of missense SNVs was predicted using three different software: PolyPhen 2 [23], SIFT [24] and VEP tool [25]. The conservation score for every amino acid position in NEU1 was calculated using ScoreCons [26] and the multiple alignment provided in [4]. According to gene data from RefSeq database, we also divided the identified variants based on the exon in which they are located. Based on NEU2 crystal structure from RCSB database (1VCU) and the predicted structure of NEU1 protein derived by homology modeling, we also grouped genetic variants in three categories, namely strand, helix or other (comprising turn, bend and disordered portion), according to the position in which they fall in the generated 3D model. Significance of enrichment for overall SNVs or non synonymous variants was tested in every category applying a binomial test. For exon categories the total dimension of the gene was considered for calculation, while for secondary structure categories the dimension of the coding sequence (CDS) was used. When testing enrichment of non synonymous variants we used the number of non synonymous sites instead of the total number of bases to calculate the success probability and the number of trials for each category.

As already mentioned, data from 1000G and ESP6500 databases allow for the study of allele frequencies and genotypes in global dataset as well as in defined subpopulations. American (AMR), African (AFR) and European (EUR) subpopulations were considered in the 1000G dataset, as these 3 groups represent the better overlap with the 2 subpopulations of African American (AA) and European American (EA), present in the ESP6500 dataset. We analyzed data from these subpopulations and identified as population specific those variants with a MAF (Minor Allele Frequency) >5% in one of the considered subpopulations and MAF<1% in all the others.

From the missense SNVs identified in *NEU1* gene, we selected a set of novel candidate mutations for subsequent functional studies according to this parameters: MAF<1%, present only in heterozygous state, not already reported in HGMD [13] database, predicted as damaging by at least 2 out of the 3 functional prediction algorithms described above. Location of the relevant variants identified within the *NEU1* mRNA sequence is shown in Figure S2.

### NEU1 structure prediction and analysis

Prediction of the human NEU1 3D structure was obtained by homology modeling using I-Tasser [27] and the known crystal structure of human NEU2 (1VCU). Refinement of the secondary structure elements, i.e. beta-strands and alpha-helices, has been carried out based on MUSCLE alignment and the positions of these structural elements in human NEU2. The already known sialidosis mutations together with our new candidates were placed on the predicted structure in order to assess their possible impact on the structural conformation of human NEU1, as well as the position of the corresponding side chains (inner core or surface of the polypeptide). All structure manipulations have been carried out using PyMol (The PyMOL Molecular Graphics System, Version 1.5.0.1 Schrödinger, LLC).

### Generation of NEU1 and PPCA constructs

Complete CDS of human *NEU1* (NM_000434.3) and *CTSA* (alias *PPCA*, NM_001127695.1) genes were amplified from 50 ng of human liver cDNA using primers containing the appropriate restriction sites (see Table S1 in File S1) and cloned into pIRES-hrGFP-1a and pcDNA3.1-Myc/HIS-a vectors, respectively. Both genes were cloned with their own stop codon, so that the expressed proteins do not contain any tag epitope. The pIRES-hrGFP-1a vector promotes the expression of a polycistronic mRNA encoding the gene of interest together with a humanized form of GFP, which allows for visual assessment of transfection efficiency in mammalian cells. The 9 single base substitutions selected for functional studies were inserted into the *NEU1* wild-type CDS using the QuickChange II SiteDirected Mutagenesis kit (Agilent) and the desired mutagenesis primer pairs (see Table S1 in File S1), according to manufacturer protocol. Nine mutated constructs were generated: pIRES-G88A-hrGPF, pIRES-L90F-hrGPF, pIRES-P210A-hrGPF, pIRES-V217A-hrGPF, pIRES-T222M-hrGPF, pIRES-D234N-hrGPF, pIRES-G248S-hrGPF, pIRES-G252S-hrGPF, pIRES-S351R- hrGPF.

### Cell culture and transfection

COS7 cells (cell line acquired from ATCC) were cultured in DMEM (Dulbecco's modified Eagle's medium) (EuroClone) containing 4 mM l-glutamine, 100 units/ml penicillin, 100 µg/ml streptomycin and 10% (v/v) fetal bovine serum and were maintained at 37°C and 5% CO_2_ in a humidified incubator. Cells were co-transfected with pcDNA3.1 PPCA and pIRES hrGPF-1a vector containing either wild-type or mutagenized *NEU1* CDS (constructs described above) in a 1∶1 ratio (mol∶mol). Transfections were performed in serum-free medium (OptiMEM) employing FuGENE HD (Promega) as transfectant agent. After 24 h transfection the medium was changed and after further 24 h cells were harvested. In all experiments, transfection efficiency of NEU1 constructs was assessed by the ratio of GFP positive cells on total cells counted in 3 different areas of the culture dish.

### Confocal microscopy analysis

COS7 cells were seeded on to glass coverslips and after 24 h were co-transfected with pcDNA3.1 PPCA and pIRES NEU1-hrGPF-1a (wild-type or V217A, D234N mutants) as indicated above. After 24 h transfection the medium was changed and after further 24 h cells were washed three times with PBS containing 1 mM MgCl_2_ and 1 mM CaCl_2_ (PBS^++^), fixed and permeabilized with cold methanol for 10 min and acetone for 1 min. After three washes and saturation with 1% BSA in PBS^++^ (PBS^++^/BSA), glass coverslips were incubated with the following primary antibody: rabbit anti-NEU1, Rockland 1∶500, mouse anti-LAMP1, BD Pharmigen 1∶200, mouse anti-PDI, STRESSGEN 1∶200. Subsequently, cells were washed and incubated with the following secondary antibody: Donkey anti-rabbit Alexa-555 and goat anti-mouse Alexa-405 1∶300 (Molecular Probes, Invitrogen) diluted in PBS^++^/BSA. Finally, specimens were mounted using Dako Cytomation Fluorescent Mounting Medium and analyzed using the confocal system LSM-510 META (Carl Zeiss). Images were processed with LSM Image Browser (Carl Zeiss) and Adobe Photoshop software.

### Western-blot and densitometric analysis

Proteins samples (10 µg) were separated by SDS/10% PAGE and transferred to a Hybond-P PVDF membrane (GE Healthcare). Membranes were then blocked, washed and incubated with the following primary antibody: rabbit anti-NEU1 1∶500 (Rockland), rabbit anti-PPCA 1∶250 (Rockland) and mouse anti-α-tubulin 1∶16000 (Sigma). Detection of the immunocomplexes was performed using appropriate HRP (horseradish peroxidase)-conjugated secondary antibodies and an enhanced chemiluminescence-based system (SuperSignal West Pico Chemiluminescent Substrate; Pierce). Focusing on the NEU1 mutants V217A and D234N, a more detailed analysis was conducted on PPCA protein. All the biological replicates from each NEU1 WT, V217A and D234N were loaded on a single SDS-PAGE gel, together with a common reference loaded in all the three gels to allow for comparison of protein signals between the three different experiments. The common reference was obtained pooling together 5 µl from all the cell extracts from wild-type NEU1 co-transfection experiments. Western-blots with anti-PPCA and anti-α-tubulin antibodies were then performed as described above, followed by densitometric analysis using GelPro 3.1 software (Media Cybernetics). We then normalized values obtained for PPCA using the correspondent α-tubulin signals. Normalized PPCA signals for WT, V217A and D234N samples were finally divided for the normalized PPCA signal of the corresponding reference sample, resulting in comparable values of PPCA protein level. The mean of relative normalized PPCA signals was 1.04 with a confidence interval 95% of 0.62. Samples showing values below the confidence interval were discarded from sialidase activity study.

### Sialidase activity assay and protein determination

The enzymatic activity in total cell lysates was determined as previously described [1] using 1 mM 4MU-NeuAc (4-methylumbelliferyl-*N*-acetyl-α-d-neuraminic acid, Sigma) as substrate. Assays were performed in triplicate with 10 µl sample volume in a final volume of 30 µl. Samples were incubated at 37°C for 30 min. Reactions were stopped using 0.2 M Glycine/NaOH pH 10.8 and activity was measured using Jasco FP-770 Spectrofluorimeter. Fluorescent intensity was referred to a standard concentration curve of 4MUB (4-methylumbelliferone). Protein concentration was determined by dye-binding assay (Coomassie Protein Assay Reagent, SIGMA) according to manufacturer's manual. A standard two-tailed t-test was calculated for every mutant taking into account all the replicated experiments to assess the significance of enzymatic activity variation compared to the wild-type NEU1. When calculating the enzymatic activity of V217A and D234N mutant proteins, 3 samples with significant lower level of normalized PPCA protein (determined as described above) were discarded resulting in a final dataset of 5 biological replicates for each mutant.

## Results

### Genetic variations in *NEU1* gene

Searching for single nucleotide variants (SNVs) in the 1000G and ESP6500 databases, we retrieved a total of 63 SNVs in *NEU1* gene. Among the 44 SNVs within the gene exons, 10 are located in the UTRs regions and 34 in the coding DNA sequence (CDS). Considering the CDS of the *NEU1* gene, the mutational rate resulted in 0.034 with a dN/dS ratio of 0.29. A summary of all the SNVs present in the *NEU1* gene, grouped by functional categories, is given in Table 1.

**Table 1:** Summary information on *NEU1* gene and related SNVs.

cDNA (bp)	CDS (bp)	Protein (aa)	Intronic variants	Exonic variants				Mutation rate	dN/dS
				Missense	Synonymous	LoF	UTR		
2088	1248	415	19	16	17	1	10	0.034	0.29

*NEU1* cDNA sequence refers to NM_000434.3, protein sequence refers to NP_000425.1. LoF, loss of function; UTR, located in the untranslated regions. dN/dS measures the level of evolutionary constrain acting on the *NEU1* gene (see Methods).

Since we are mainly interested in variants affecting the function of the NEU1 enzyme, as possible pathological alleles in sialidosis, we focused our further analysis only on SNVs occurring in the CDS of this gene. Overall, we identified 17 synonymous variants, 16 missense substitutions and 1 small indel generating a frameshift. We failed to identify nonsense substitutions as well as variants altering a splicing site. The synonymous variants are listed in Table 2 with detailed annotations, while the 16 missense and the single frameshift variants identified are reported in Table 3.

**Table 2:** Synonymous variants in *NEU1* CDS.

Genomic coordinate (hg19)	dbSNP 139 ID	Nucleotide change in CDS	GERP score
6:31830455	rs141879244	c.99G>A	0.15
6:31829190	rs115468005	c.390T>C	−10.3
6:31829178	rs142833447	c.402C>T	−10.3
6:31829172	rs41267074	c.408G>A	1.11
6:31829148	rs114405905	c.432C>T	2.3
6:31829112	rs143737826	c.468C>T	1.3
6:31829094	rs115588976	c.486C>T	1.6
6:31828986	rs140168128	c.594T>C	−3.17
6:31828366	rs145784816	c.648C>T	−2.83
6:31828348	rs370667977	c.666G>A	−3.46
6:31828315	rs188562197	c.699C>T	−8.05
6:31828288	rs376599274	c.726C>T	−9.19
6:31828267	rs114143271	c.747C>T	2.37
6:31827904	rs201379546	c.936G>A	3.6
6:31827901	rs149992593	c.939C>T	−7.22
6:31827637	rs150864071	c.1107C>A	4.46
6:31827505	rs114618932	c.1239G>A	−3.45

*NEU1* cDNA sequence refers to NM_000434.3. GERP (Genomic Evolutionary Rate Profiling) score measures the level of conservation for the indicated nucleotide (see Methods).

**Table 3:** Missense and LoF variants in *NEU1* CDS.

Genomic coordinate	DbSNP 139 ID	Variants in CDS	Effect on protein	Structure element	GERP score	Impact prediction	Global % MAF (ESP6500/1000G)
6:31829865	rs34712643	c.263G>C*	p.G88A	loop	5.46	D	2.6/2.0
6:31829860	rs374556080	c.268C>T*	p.L90F	β-sheet	5.46	D	0.01/nd
6:31829044	rs150302766	c.536T>C	p.V179A	loop	5.7	B	0.01/nd
6:31828391	rs375104221	c.623G>A	p.R208Q	loop	1.7	B	0.02/nd
6:31828386	rs151177689	c.628C>G*	p.P210A	loop	5.93	D	0.01/nd
6:31828365	rs28940583	c.649G>A†	p.V217M	loop	5.84	D	0.03/0.05
**6:31828364**	**rs146850952**	**c.650T>C** *	**p.V217A**	**loop**	**5.84**	**D**	**0.01/nd**
6:31828349	rs201684013	c.665C>T*	p.T222M	β-sheet	5.84	D	0.01/nd
**6:31828314**	**rs143868999**	**c.700G>A** *	**p.D234N**	**loop**	**4.45**	**D**	**0.01/nd**
6:31828287	rs104893983	c.727G>A†	p.G243R	loop	5.32	D	0.01/nd
6:31828272	rs373311653	c.742G>A*	p.G248S	loop	5.32	D	0.01/nd
6:31828260	rs145177628	c.754G>A*	p.G252S	loop	5.32	D	0.02/nd
**6:31828246**		**c.759_760insGA**	**p.P254fs**	**loop**			**0.01/nd**
6:31828005	rs368320390	c.835G>A	p.A279T	β-sheet	1.38	B	0.01/nd
6:31827960	rs190549838	c.880C>A†	p.R294S	loop	5.1	D	nd/0.0
6:31827691	rs377573360	c.1053C>G*	p.S351R	loop	3.64	D	0.01/nd
6:31827674	rs139301823	c.1070G>A	p.R357Q	loop	0.38	B	0.08/nd

*  = variants selected for in vitro functional studies;

† = variants already annotated as pathological mutations in HGMD [13].

The two missense variants identified as putative pathogenic mutations and the frameshift variant are shown in bold. Impact prediction summarizes results from the PolyPhen2, SIFT and VEP tools: D, damaging; B, benign or tolerated. GERP (Genomic Evolutionary Rate Profiling) score measures the level of conservation for the indicated nucleotide (see Methods). MAF, minor allele frequency; nd, not reported in the corresponding database. *NEU1* cDNA sequence refers to NM_000434.3, protein sequence refers to NP_000425.1.

Using the information available from 1000G and ESP6500 databases, we also retrieved allele frequencies and genotype counts for the 16 missense variants and the single frameshift variant reported in *NEU1* gene (Tables S2 and S3 in File S1). All *NEU1* missense variants are rare (MAF<1%) and present only in heterozygous state, with the exception of the missense variant c.263G>C, resulting in the p.G88A amino acid substitution. This variant has a MAF of 2 and 2.6% and is present in homozygous state in 1 and 7 individuals in the global populations from 1000G and ESP6500, respectively.

Interestingly, this SNV shows a population specific distribution, being common only in the African population from the 1000G dataset (MAF 10%) and in African-American population from ESP6500 dataset (MAF 7.2%). In the other population subgroups it shows MAF<1%, and it is present only in heterozygous state (see Tables S2 and S3 in File S1).

**Figure 1:**
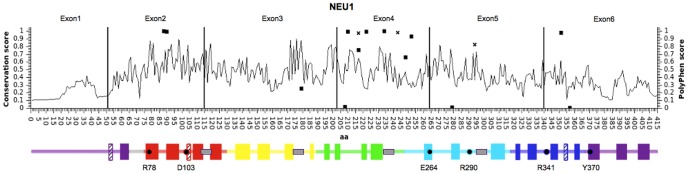
Distribution of variants identified in NEU1. Schematic representation of NEU1 protein, with amino acid position on the X axis. Black vertical lines divide the 6 exons of *NEU1* gene. Line graph indicates the conservation score for each amino acid position as calculated by ScoreCons. The identified missense variants are represented as squares, with Y values corresponding to PolyPhen score. The missense variants already identified as pathological alleles in sialidosis are represented as diagonal crosses. Below the graph, schematic representation of NEU1 structural elements: full color blocks represent beta-strand elements, while crossed blocks represent helices. Color code identifies the 6 blades of the β-propeller structure as described in [35]. Gray boxes represent the Asp-box elements and essential catalytic residues are indicated with dots and their residue number. Positions of all elements are relative to the amino acid position in the graph above.

Based on the NEU2 protein structure and the structural models predicted for the other human sialidases, we grouped the NEU1 variants according to secondary structural elements (Table 4 and Figure 1). Only structural elements connecting antiparallel beta-strands resulted significantly enriched in missense variants (p 0.011). Based on the distribution of the identified SNVs within the *NEU1* gene and the corresponding protein (Table 5 and Figure 1), we found a significant enrichment of variants (15) in exon 4 (p 7.34 E-06), while the number was significantly lower in exon 6 (p 0.002). Figure 1 also shows the conservation score (calculated by ScoreCons) for every amino acid position and a schematic representation of the predicted secondary structure of the NEU1 protein. Notably, most of the identified missense variants are located at low conserved positions within the regions connecting the antiparallalel beta-strands.

**Table 4:** Distribution of SNVs in secondary structural elements of NEU1 protein.

α-helix			β-sheet			Loop		
bp	syn	mis	bp	syn	mis	bp	syn	mis
27	0	0 (0.794)	516	9	3 (0.158)	702	8	12 (0.011)

Numbers in brackets represent the p-value associated to the enrichment of SNVs in the corresponding category calculated as described in Methods. Classification of variants: mis, missense; syn, synonymous.

**Table 5:** Distribution of SNVs in the exon of *NEU1* gene.

Exon1			Exon2			Exon3			Exon4			Exon5			Exon6		
(0.162)			(0.140)			(0.086)			(7.34 E-06)			(0.17)			(0.002)		
bp	Syn/UTR	mis	bp	syn	mis	bp	syn	mis	bp	syn	mis	bp	syn	mis	bp	Syn/UTR	mis
289	6	0	193	0	2	263	7	1	183	5	9	223	2	3	894	7	1

Based on *NEU1* cDNA sequence NM_000434.3. Numbers in brackets represent the p-value associated to the enrichment of SNVs in the corresponding exon calculated as described in Methods. Classification of variants: mis, missense; syn, synonymous; UTR, located in the untranslated regions.

### Identification of candidate variants in NEU1 for *in vitro* studies

Among all *NEU1* gene variants, we selected 8 missense SNVs, predicted to be potentially damaging, for in vitro functional studies. These SNVs are rare (MAF<1%), present only in heterozygous state and not previously known as causative mutations in sialidosis. To this group of variants we added the c.263G>C SNV described above, because of its population-specific distribution. Thus, a total of 9 variants (marked with * in Table 3) were subjected to *in vitro* functional studies.

**Figure 2:**
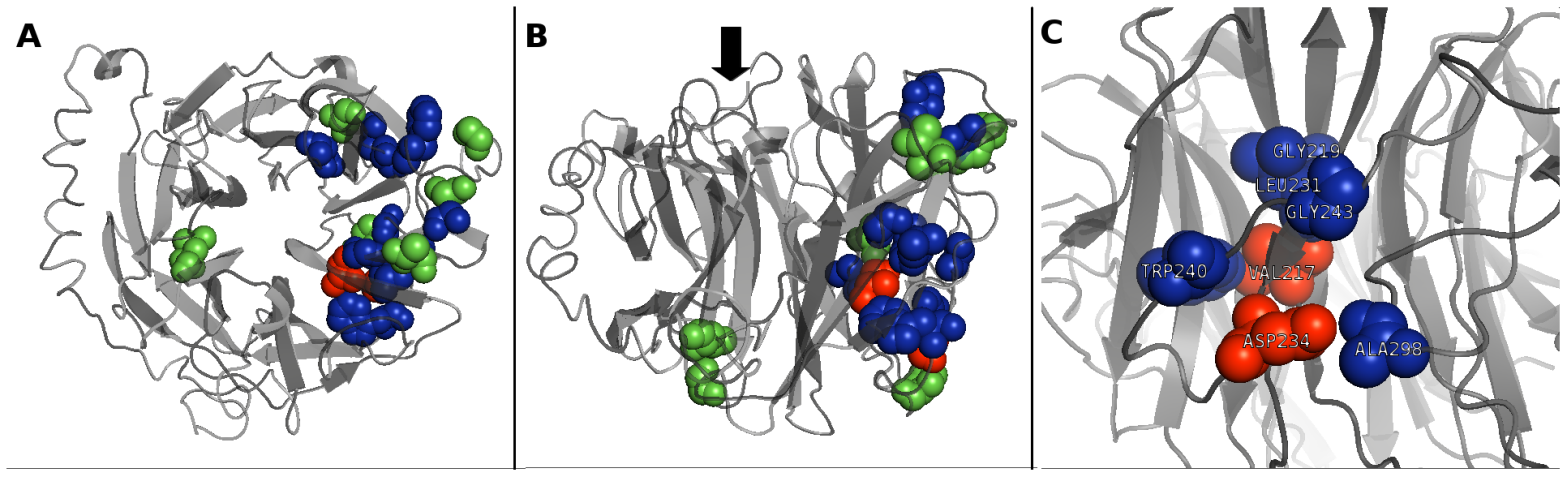
NEU1 structure analysis and localization of the 9 candidate missense mutations. Top (A) and lateral (B) views of the structural model of NEU1 protein. The two amino acids V217 and D234, identified in this work as affected by new putative pathological mutations, are shown in red; the 7 amino acids affected by the other candidate missense variants tested are shown in green. Other amino acids already reported as mutated in sialidosis and likely involved in NEU1-PPCA interaction [28] are shown in blue. The black arrow in (B) indicates the side of the catalytic crevice. (C) Detailed view of the localization of V217 and D234. These two amino acids, together with G218, L231, W240, G243 and A298 define a region of the NEU1 protein that could represent an important site in the surface interaction with PPCA partner.

We also identified a single frameshift insertion c.759_760insGA that alters the wild-type protein from amino acid 254 onward. Due to the dramatic effect of this variant on the protein, we chose not to include it in our *in vitro* assays. The 9 candidate missense variants were positioned within the NEU1 structural model to assess their impact on the protein structure and their position relative to the active site (Figure 2A and 2B). A close-up image of the position of p.V217A and p.D234N amino acid substitutions relative to other amino acids previously known to be involved in NEU1-PPCA interaction [28] is shown in Figure 2C. Of these 9 missense variants, seven affect amino acids located near the putative contact region between NEU1 and PPCA as proposed in [28]: P210, V217, T222, D234, G248, G252, S351.

### Enzymatic activity assays and immunoblotting of NEU1 mutants

**Figure 3:**
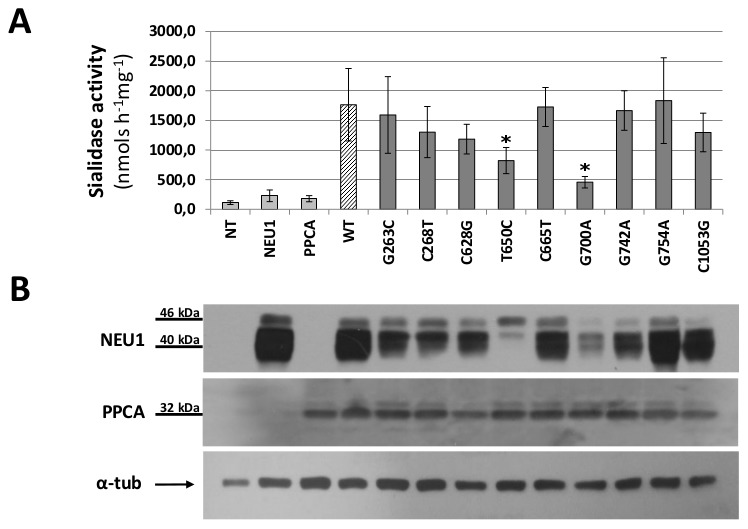
Sialidase activity assay and immunoblotting analysis. (A) Sialidase specific activity calculated for COS7 cells non transfected (NT), transfected with NEU1 wt alone (NEU1), PPCA alone (PPCA), NEU1 wt and PPCA (WT) and NEU1 mutants and PPCA. V217A and D234N mutant proteins resulted in a significant reduction in sialidase activity (p<0.05, marked with *). (B) Immunoblotting for PPCA and NEU1 proteins in each sample. NEU1 showed the expected signals between 40 and 46 kDa, corresponding to different glycosylation states; PPCA showed the expected signal at 32 kDa, corresponding to the heavy peptide of the active form of the protein. α-tub was used for protein loading normalization. The image is representative of 3 replicate experiments.

COS7 cells were co-transfected with the wild-type and mutants *NEU1* cDNAs together with the human *PPCA* cDNA, which is essential for the efficient lysosomal compartmentalization and catalytic activation of NEU1 [2], [12]. The sialidase activity of the individual NEU1 variant proteins expressed in COS7 cells was calculated as the average of 8 independent experiments, that gave comparable transfection efficiency (22%±3), based on GFP expression (Figure 3A). Immunoblotting of transfected cell lysates with anti-NEU1 antibodies showed the expected bands of 40 and 46 kDa, reflecting differences in the extent of glycosylation of the NEU1 proteins, as previously reported [1], [29] (Figure 3B). As expected, transfection of wild-type *NEU1* or *PPCA* cDNAs alone did not result in a significant increase in sialidase activity (Figure 3A). Instead, co-transfection of wild-type NEU1 and PPCA resulted in a 16.6±5.9 fold increase in sialidase activity compared to non-transfected cells, corresponding to 1876±414 nmols h^−1^ mg^−1^. Co-transfection of all 9 NEU1 candidate variants with PPCA also led to a significant increase in sialidase activity compared to non-transfected cells. Nevertheless, the two variants c.650T>C and c.700G>A showed significantly lower levels of sialidase activity (p<0.05) than the wild-type NEU1 (Figure 3A). Immunoblotting analysis confirmed that the protein levels of these two NEU1 mutants were substantially decreased compared to the levels of the wild-type protein and the other mutants (Figure 3B). After discarding the samples with low expression levels of PPCA (figure S1), we determined that the V217A and D234N NEU1 mutants had a specific activity of 793±233 and 458±97 nmols h^−1^ mg^−1^, respectively. Thus, these two NEU1 mutants retained only 44% and 25% of the sialidase activity of the wild-type enzyme.

### Subcellular localization of NEU1 mutants V217A and D234N

**Figure 4:**
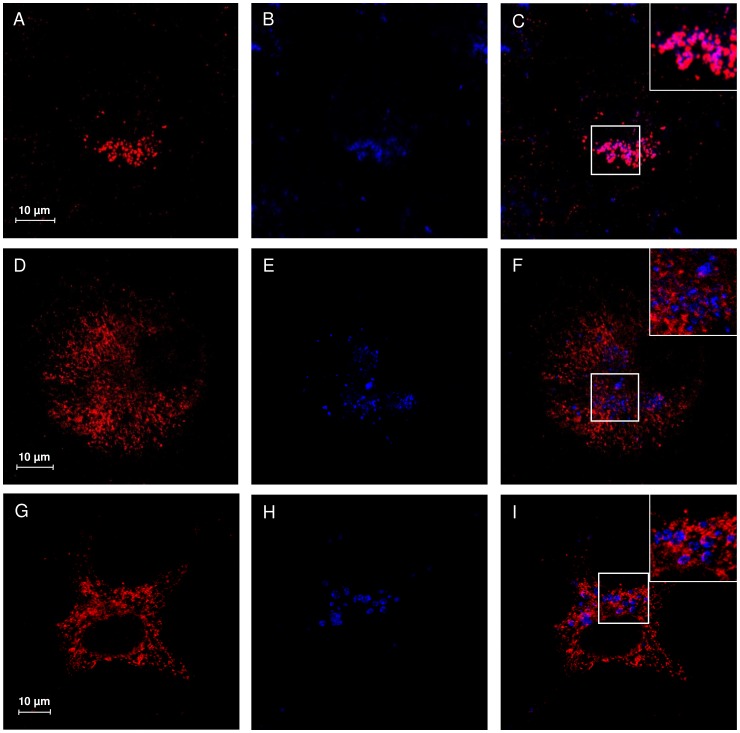
Subcellular localization study of V217A and D234N NEU1 mutant proteins. Confocal microscopy images showing the subcellular distribution of NEU1 wild-type, V217A and D234N mutants. NEU1 proteins were detected using specific rabbit anti-NEU1 antibody and revealed with Alexa-555 secondary antibody. Wild-type NEU1 labeling resulted in a vesicular pattern (A), mainly colocalizing with the lysosomal marker LAMP1 detected with mouse anti-LAMP1 and revealed with Alexa-405 secondary antibody (B, merge in C). The V217A (D) and D234N (G) mutant proteins showed a tubulo-reticular localization. Almost no colocalization between V217A and D234N mutants and LAMP1 (E–F and H–I) could be detected. Insets represent enlargement of the indicated areas.

**Figure 5:**
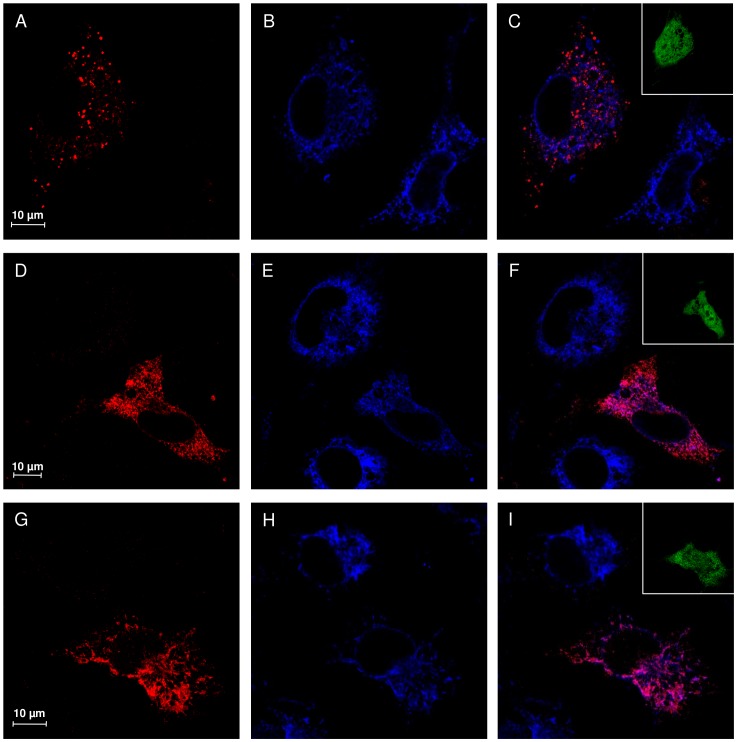
V217A and D234N NEU1 mutant proteins are accumulated in the ER. Confocal microscopy images showing the subcellular distribution of NEU1 wild-type, V217A and D234N mutants. NEU1 proteins were detected using specific rabbit anti-NEU1 antibody and revealed with Alexa-555 secondary antibody. Wild-type NEU1 labeling resulted in a vesicular pattern (A), with no evident colocalization with the ER marker PDI detected with mouse anti-PDI and revealed with Alexa-405 secondary antibody (B, merge in C). The V217A (D) and D234N (G) mutant proteins showed a tubulo-reticular localization with significant colocalization areas with PDI. Insets represent the GFP signal, indicating transfected cells. No evident leak of the green signal into the red or blue channel is detected.

Confocal microscopy analysis of COS7 cells transfected with wild-type NEU1 showed a distribution of the protein mainly organized in vesicular structures (Figure 4A), colocalizing with the lysosomal marker LAMP1 (Figure 4C). Interestingly, cells expressing either V217A or D234N NEU1 mutants showed a different subcellular distribution of these proteins, without evident colocalization with LAMP1 (Figure 4F and 4I), and a labeling that appears concentrated in reticular and filamentous structures, suggestive of the endoplasmic reticulum (Figure 4D and 4G). To confirm these results, we also analyzed the distribution of wild-type, V217A and D234N NEU1 proteins in relation to PDI as ER marker (Figure 5) and areas of significant colocalization have been found only for the two mutant forms of NEU1 (Figure 5C, F and I).

## Discussion

### Genetic variability in *NEU1* and identification of rare *NEU1* alleles

The analysis of *NEU1* genetic variability in public data from large sequencing programs allowed us to recover a total of 63 single nucleotide variants (SNVs), 44 of them in the exon regions, including 17 with potential functional impact, namely missense or loss of function (LoF) variants (Table 1). The *NEU1* gene shows a relatively small number of SNVs that alter the sequence of the encoded protein. This has to be expected considering the essential physiological role of this enzyme, whose impaired activity leads to sialidosis, a severe mendelian disease. The high level of negative selective pressure acting on *NEU1* gene is also confirmed by a low dN/dS value [30]. SNVs appear to distribute unevenly throughout the *NEU1* gene (Table 5 and Figure 1), with exon 4 significantly enriched in variants. Intriguingly, this exon encodes a region of the protein near the loop corresponding to the highly variable region previously identified in NEU3 and NEU4 [4], supporting the hypothesis that this portion of the sialidase proteins is more tolerant to amino acid substitutions. Moreover, 13 out of 16 missense substitutions identified in this study fall in regions connecting antiparallel beta-strands of NEU1 predicted protein structure (Table 4). Overall, these results confirm previous studies, demonstrating that loop regions are less conserved and particularly subjected to amino acid substitution in sialidase protein family [4], [31].

While most of the functional variants in the *NEU1* gene are rare, with MAF<1%, we also identified one more common missense substitution (c.263G>C) with global MAF>2%, that is restricted to the African and African American sub populations (MAF>7%). The high allele frequency observed only in a single sub population suggests a founder effect, with the SNV arising in an ancestor of the subgroup and then spreading within it. Enzymatic assays showed that this variant does not alter the level of enzyme activity and it is unlikely that it can influence its substrate specificity or kinetic parameters, given its position on the opposite side of the catalytic crevice (Figure 2A and 2B).

Overall, considering the already known pathogenic variants, together with the two new missense mutations (c.650T>C and c.700G>A) and the frameshift insertion c.759_760insGA identified in this study, we found a total of 6 pathogenic alleles in a population of 7595 individuals. This results in a carrier frequency of 1 every 1266 subjects, close to the value of 1 every 1025 expected for sialidosis given the case of Hardy-Weinberg equilibrium and the estimated incidence of 1/4,200,000 [7].

Given the large number of subjects enrolled in the 1000G and the ESP6500 sequencing projects, the analysis of these databases is effective to reveal rare variants in known disease-causing genes [20], [32] that could represent undetected pathological alleles present in the population [33]. In this study, we searched these databases for SNVs in *NEU1* gene that led to reduced activity of the enzyme.

Since the two databases contain data from healthy subjects in their middle age, we presumed that both known and new causative mutations would be present as rare alleles only in the heterozygous state. Applying the prioritization strategy described in methods, we identified 9 missense variants with MAF≤0.1% (marked with * in Table 3) not previously reported as associated with sialidosis in HGMD database [13], as well as 3 SNVs (rs190549838, rs104893983, rs28940583) already identified in patients with sialidosis, with the latter two having an allele frequency of 0.07 and 0.3%, respectively. The fact that MAF value for rs190549838 was equal to 0 is due to a known issue in the 1000G dataset. We also identified a novel single frameshift insertion c.759_760insGA that alters the open reading frame of the wild-type protein from amino acid 254. Even if we did not test it with functional *in vitro* assays, this variant can be considered a pathological mutation, since it severely affects NEU1 peptide sequence, and also removes essential catalytic residues R341 and Y370 [2], [3].

None of the 9 missense variants described above affects amino acids which are located near the active site in the generated NEU1 structure model. However, 7 of these residues (P210, V217, T222, D234, G248, G252, S351) are positioned in a region that is possibly involved in the NEU1-PPCA interaction, according to structural modeling of NEU1 pathological mutations [28] (Figure 2A and 2B).

We further characterized the 9 newly identified missense variants for their impact on lysosomal sialidase enzymatic activity and subcellular localization. The individual NEU1 mutants were co-expressed with the human *PPCA* cDNA, which is essential for the proper compartmentalization and catalytic activation of wild-type NEU1 [2], [12]. We found that the majority of the analyzed NEU1 variants maintain the properties of the wild-type enzyme, are correctly glycosylated and thus potentially as active as the wild-type enzyme in our expression model [29]. To avoid bias in sialidase activity, only experiments with homogeneous transfection efficiency of the NEU1 and PPCA constructs were subjected to enzymatic activity assays. This study allowed the identification of two variants, namely c.650T>C (p.V217A) and c.700G>A (p.D234N), showing significantly lower sialidase activity compared to the wild-type protein (Figure 3A).

### Characterization of the V217A and D234N mutant proteins

A precise measurement of the sialidase activity of the V217A and D234N protein variants is essential to evaluate their role as defective enzymes causative for sialidosis [1], [5], [6]. We thus decided to apply the normalization strategy described in methods to obtain a robust estimation of enzyme specific activity for the two candidate pathological mutants (Figure 3 and Figure S1).

The c.650T>C mutant showed a 44% residual activity compared to the wild-type enzyme. Even if the residual activity is still quite high compared to non-transfected cells, the mutation results in the p.V217A amino acid change, involving the same residue of the p.V217M substitution already described in a patient carrying sialidosis type I [34] and present in the HGMD database. This mutation is supposed to alter the interaction between NEU1 and PPCA, thus preventing the correct transport and maturation of the enzyme [34]. This evidence strongly suggests that also the c.650T>C mutation may affects the NEU1-PPCA interaction and could represent a pathological allele responsible for the mild form of the disease.

The c.700G>A mutant, resulting in the p.D234N amino acid change, showed a 25% residual activity compared to the wild-type enzyme. Such a strong reduction is in the range of residual activity reported for many other pathological mutations already identified in *NEU1* gene [5], [8], [9].

Based on NEU1 3D model, both p.V217A and p.D234N are located in the same protein region containing the other already known pathological mutations predicted to affect the NEU1-PPCA interaction [28] (Figure 2B). In particular the two residues V217 and D234, together with the residues G218, L231, W240, G243 and A298 define a region of the NEU1 protein that could represent an important site in the surface interaction with PPCA partner (Figure 2C). As already described in another case of sialidosis [34], both mutants showed reduced NEU1 protein levels by Western-blot analysis (Figure 3B) which correlate with the reduction in sialidase activity. Moreover, their subcellular localization resulted altered compared to the wild-type protein and the mutant NEU1 proteins showed almost no signal in vesicular structures compatible with the lysosomal compartment (Figure 4). Instead, V217A and D234N mutant proteins were mainly localized in the ER (Figure 5), supporting the idea of an altered protein trafficking that probably results in protein degradation. Overall, Western-blot and subcellular localization data suggest that p.V217A and p.D234N substitutions could play a role in the NEU1-PPCA interaction/recognition, a well known pivotal step for the correct localization of the sialidase enzyme [2], [12], [28] and/or cause a reduced stability of the protein.

In this perspective, both mutants represent new putative pathological mutations causative for sialidosis, at least in the late onset form of the disease. Given the emerging role of NEU1 in several multifactorial diseases [14]–[19], the identification of new protein variants with altered enzymatic activity could be of interest for future studies aimed at investigating the involvement of NEU1 functional variants in the pathogenesis of complex disorders.

## Conclusions

The use of genomic data from large sequencing programs is an effective strategy to investigate genetic variability in humans. This study led to the identification of previously unknown *NEU1* alleles diffused in the actual population. These data are useful for future functional studies on human sialidase enzymes. *In vitro* functional studies on variants occurring in *NEU1* gene also led to the identification of two new putative disease-causing mutations responsible for sialidosis. In summary, we identified a total of 3 known and 3 novel putative sialidosis disease alleles in a cohort of 7595 individuals, a number compatible with the estimated prevalence of the disease. The discovery of rare variant based on large genomic dataset, combined with well established functional test in cellular models, prove to be an effective strategy to identify new causative mutations in known disease genes and to assess their functional impact.

## Supporting Information

Figure S1 **Evaluation of PPCA protein level in cells transfected with V217A and D234N mutant proteins.** Immunoblotting analysis on NEU1 wt, V217A and D234N cell extracts deriving from different experiments. The amount of PPCA protein was evaluated in each of the 8 biological replicates and only the 5 samples showing an homogeneous level of the protein were considered (see Methods). Numbers show the relative normalized amount of PPCA protein from densitometric analysis, calculated as described in Methods.
(TIF)

Figure S2 **Nucleotide variants identified in** ***NEU1*** **cDNA.** The complete sequence of *NEU1* cDNA (NM_000434.3) is reported, with ORF in uppercase. Numbering of relevant nucleotides, starting from the ATG, is reported in superscript. Both starting ATG and stop codon TGA are underlined. The functional nucleotide variants (missense or LoF) are reported in bracket (reference base/variant allele): in magenta, already known disease mutations for sialidosis; in red, the 3 variants identified in this work as new putative pathological alleles; in green, the other variants tested by functional assays; in light blue, the remaining untested variants.
(PDF)

File S1 **Supporting tables.** **Table S1, Oligonucleotide primers used for PCR amplification and mutagenesis. Table S2, Allele frequencies and genotype counts for NEU1 missense variants from ESP6500 database. Table S3, Allele frequencies and genotype counts for NEU1 missense variants from 1000G database.**
(PDF)
