# Supplementary material for: In Silico Identification of New Putative Pathogenic Variants in the Neu1 Sialidase Gene Affecting Enzyme Function and Subcellular Localization
Source: PLoS One. 2014 Aug 25;9(8):e104229. doi: 10.1371/journal.pone.0104229 (PMC4143216; doi:10.1371/journal.pone.0104229)
Supplement: Figure S2 — Nucleotide variants identified in NEU1 cDNA. The complete sequence of NEU1 cDNA (NM_000434.3) is reported, with ORF in uppercase. Numbering of relevant nucleotides, starting from the ATG, is reported in superscript. Both starting ATG and stop codon TGA are underlined. The functional nucleotide variants (missense or LoF) are reported in bracket (reference base/variant allele): in magenta, already known disease mutations for sialidosis; in red, the 3 variants identified in this work as new putative pathological alleles; in green, the other variants tested by functional assays; in light blue, the remaining untested variants. (PDF) [file pone.0104229.s002.pdf]

>NM_000434.3
gagctacttgaagaccaattagagtccgggaagcgcggcggggcctccagaccggggcgggcttaagggtgacatctgcg
ctttaaagggtccgggtcagctgactcccgactctgtggagtctagctgccagggtcgcggcagctgcggggagag[1]ATG
ACTGGGGAGCGACCCAGCACGGCGCTCCCGGACAGACGCTGGGGGCCGCGGATTCTGGGCTTCTGGGGAGGCTGTAGGGT
TTGGGTGTTTGCCGCGATCTTCCTGCTGCTGTCTCTGGCAGCCTCCTGGTCCAAGGCTGAGAACGACTTCGGTCTGGTGC
AGCCGCTGGTGACCATGGAGCAACTGCTGTGGGTGAGCGGGAGACAGATCGGCTCAGTGGACACCTTCCGCATCCCGCTC
ATCACAGCCACTCCGCGGG[263](G>C)CACT[268](C>T)TTCTCGCCTTTGCTGAGGCGAGGAAAATGTCCTCATCCGATGA
GGGGGCCAAGTTCATCGCCCTGCGGAGGTCCATGGACCAGGGCAGCACATGGTCTCCTACAGCGTTCATTGTCAATGATG
GGGATGTCCCCGATGGGCTGAACCTTGGGGCAGTAGTGAGCGATGTTGAGACAGGAGTAGTATTTCTTTTCTACTCCCTT
TGTGCTCACAAGGCCGGCTGCCAGGTGGCCTCTACCATGTTGGTATGGAGCAAGGATGATGGTG[536](T>C)TTCCTGGAG
CACACCCCGGAATCTCTCCCTGGATATTGGCACTGAAGTGTTTGCCCCTGGACCGGGCTCTGGTATTCAGAAACAGC[623](
G>A)GGAG[628](C>G)CACGGAAGGGCCGCCTCATC[649](G>A)[650](T>C)GTGTGGCCATGGGA[665](C>T)GCTGGAGCGGG
ACGGAGTCTTCTGTCTCCTCAGC[700](G>A)ATGATCATGGTGCCTCCTGGCGCTAC[727](G>A)GAAGTGGGGTCAGC[742](
G>A)GCATCCCCTAC[754](G>A)GTCA[759](G>GGA)CCCAAGCAGGAAAATGATTTCAATCCTGATGAATGCCAGCCCTAT
GAGCTCCCAGATGGCTCAGTCGTCATCAAT[835](G>A)CCCGAAACCAGAACAACTACCACTGCCACTGCCGAATTGTCCT
C[880](C>A)GCAGCTATGATGCCTGTGATACACTAAGGCCCCGTGATGTGACCTTCGACCCTGAGCTCGTGGACCCTGTGG
TAGCTGCAGGAGCTGTAGTCACCAGCTCCGGCATTGTCTTCTTCTCCAACCCAGCACATCCAGAGTTCCGAGTGAACCTG
ACCCTGCGATGGAGCTTCAG[1053](C>G)AATGGTACCTCATGGC[1070](G>A)GAAAGAGACAGTCCAGCTATGGCCAGGCC
CCAGTGGCTATTCATCCCTGGCAACCCTGGAGGGCAGCATGGATGGAGAGGAGCAGGCCCCCCAGCTCTACGTCCTGTAT
GAGAAAGGCCGGAACCACTACACAGAGAGCATCTCCGTGGCCAAAATCAGTGTCTATGGGACACTCTGAgctgtgccact
gccacaggggtattctgccttcaggactctgccttcaggaacacgggtctgtagagggtctgctggagacgcctgaaaga
cagttccatcttcctttagactccagccttggcaaaatcaccttccctttaccagggaaatcacttcctttaggactgaa
agctaggcgtcctctcccacaaaaaagtcctgccctcatctgagaatactgtctttccatatggctaagtgtggccccac
caccctctctgccctcccgggacattgattggtcctgtcttgggcaggtctagtgagctgtagaattgaatcaatgtgaa
ctcagggaactggggaaggctgagcctcctctttggtgttgcggtaagataaccgacagggctggtgaaagtccccagat
ggcaggatatttggtttcagagtaaggactaggtgcaccaccatgactgactatcaatcaaaatgtttgtaacttaaaat
ttttaatgaaggataatgaatatttgtagagtctctatggttctgtcaatgcacatcttcgtgtctgttttcctcatgta
tccttgtgagcctgggtgagttctggggagagacctgatgtgcgtactgcctgtgaaaatctgactttggcaaatcaaat
cctcttttccttttgaaaaaaaaaaaaaaaaaa
