# Supplementary material for: In Silico Identification of New Putative Pathogenic Variants in the Neu1 Sialidase Gene Affecting Enzyme Function and Subcellular Localization
Source: PLoS One. 2014 Aug 25;9(8):e104229. doi: 10.1371/journal.pone.0104229 (PMC4143216; doi:10.1371/journal.pone.0104229)
Supplement: File S1 — Supporting tables. Table S1, Oligonucleotide primers used for PCR amplification and mutagenesis. Table S2, Allele frequencies and genotype counts for NEU1 missense variants from ESP6500 database. Table S3, Allele frequencies and genotype counts for NEU1 missense variants from 1000G database. (PDF) [file pone.0104229.s003.pdf]

Table S1. Oligonucleotide primers used for PCR amplification and mutagenesis

Name	Sequence
NEU1_EcoRI_F	CGGAATTCCGCCACCATGACTGGGGAGCGACCCAGC
NEU1_SalI_R	CGGTCGACTCAGAGTGTCCCATAGACACTGA
NEU1_G263C_F	GCCACTCCGCGGGcCACTCTTCTCGCC
NEU1_G263C_R	GGCGAGAAGAGTGgCCCGCGGAGTGGC
NEU1_C268T_F	CCGCGGGGCACTtTTCTCGCCTTTGC
NEU1_C268T_R	GCAAAGGCGAGAAaAGTGCCCCGCGG
NEU1_C628G_F	GAAACAGCGGGAGgCACGGAAGGGCCG
NEU1_C628G_R	CGGCCCTTCCGTGcCTCCCGCTGTTTC
NEU1_T650C_F	GGCCGCCTCATCGcGTGTGGCCATGGG
NEU1_T650C_R	CCCATGGCCACACgCGATGAGGCGGCC
NEU1_C665T_F	CGTGTGTGGCCATGGGAtGCTGGAGCGGGACG
NEU1_C665T_R	CGTCCCGCTCCAGCaTCCCATGGCCACACACG
NEU1_G700A_F	CTGTCTCCTCAGCaATGATCATGGTGC
NEU1_G700A_R	GCACCATGATCATtGCTGAGGAGACAG
NEU1_G742A_F	GGAAGTGGGGTCAGCaGCATCCCCTACG
NEU1_G742A_R	CGTAGGGGATGCtGCTGACCCCACTTCC
NEU1_G754A_F	GCGGCATCCCCTACaGTCAGCCCAAGCAG
NEU1_G754A_R	CTGCTTGGGCTGACtGTAGGGGATGCCGC
NEU1_C1053G_F	CGATGGAGCTTCAGgAATGGTACCTCATGGCG
NEU1_C1053G_R	CGCCATGAGGTACCATTcCTGAAGCTCCATCG
PPCA_EcoRI_F	CGGAATTCCGCCACCATGATCCGAGCCGCGCCGCCG
PPCA_XbaI_R	GCTCTAGATCAGTATGGCTGCTTGTTCAGGAAGC

Restriction sites are underlined and mutated bases are shown in lower case.

Table S2. Allele frequencies and genotype counts for *NEU1* missense variants from ESP6500 database

**Genomic coordinate**	**ref/alt**	**MAF (%)**			**Genotypes count**								
		**EA**	**AA**	**Global**	**EA**			**AA**			**Global**		
6:31827674	C/T	0.0554	0.1325	0.083	TT=0	TC=3	CC=2705	TT=0	TC=4	CC=1505	TT=0	TC=7	CC=4210
6:31827691	G/C	0.0000	0.0331	0.0119	CC=0	CG=0	GG=2709	CC=0	CG=1	GG=1509	CC=0	CG=1	GG=4218
6:31828005	C/T	0.0000	0.0331	0.0118	TT=0	TC=0	CC=2709	TT=0	TC=1	CC=1510	TT=0	TC=1	CC=4219
6:31828246	C/CCT	0.0000	0.0234	0.0080	A1A1=0	A1R=0	RR=4127	A1A1=0	A1R=1	RR=2132	A1A1=0	A1R=1	RR=6259
6:31828260	C/T	0.0233	0.0000	0.0154	TT=0	TC=2	CC=4298	TT=0	TC=0	CC=2203	TT=0	TC=2	CC=6501
6:31828272	C/T	0.0116	0.0000	0.0077	TT=0	TC=1	CC=4299	TT=0	TC=0	CC=2203	TT=0	TC=1	CC=6502
6:31828287	C/T	0.0116	0.0000	0.0077	TT=0	TC=1	CC=4299	TT=0	TC=0	CC=2203	TT=0	TC=1	CC=6502
6:31828314	C/T	0.0116	0.0000	0.0077	TT=0	TC=1	CC=4299	TT=0	TC=0	CC=2203	TT=0	TC=1	CC=6502
6:31828349	G/A	0.0116	0.0000	0.0077	AA=0	AG=1	GG=4299	AA=0	AG=0	GG=2203	AA=0	AG=1	GG=6502
6:31828364	A/G	0.0116	0.0000	0.0077	GG=0	GA=1	AA=4299	GG=0	GA=0	AA=2203	GG=0	GA=1	AA=6502
6:31828365	C/T	0.0000	0.0908	0.0308	TT=0	TC=0	CC=4300	TT=0	TC=4	CC=2199	TT=0	TC=4	CC=6499
6:31828386	G/C	0.0000	0.0227	0.0077	CC=0	CG=0	GG=4300	CC=0	CG=1	GG=2202	CC=0	CG=1	GG=6502
6:31828391	C/T	0.0000	0.0681	0.0231	TT=0	TC=0	CC=4300	TT=0	TC=3	CC=2200	TT=0	TC=3	CC=6500
6:31829044	A/G	0.0185	0.0000	0.0118	GG=0	GA=1	AA=2708	GG=0	GA=0	AA=1511	GG=0	GA=1	AA=4219
6:31829860	G/A	0.0000	0.0331	0.0119	AA=0	AG=0	GG=2708	AA=0	AG=1	GG=1510	AA=0	AG=1	GG=4218
6:31829865	C/G	0.0369	7.2138	2.6073	GG=0	GC=2	CC=2706	GG=7	GC=204	CC=1300	GG=7	GC=206	CC=4006

Ref, nucleotide in the reference genome sequence (hg19); alt, alternative nucleotide. MAF, Minor Allele Frequency. Populations: EA, European American; AA, African American.

Table S3. Allele frequencies and genotype counts for *NEU1* missense variants from 1000G database

<table>
<tr><th rowspan="2">Genomic coordinate</th><th rowspan="2">ref/alt</th><th colspan="4">MAF (%)</th><th colspan="12">Genotypes count</th></tr>
<tr><th>EUR</th><th>AMR</th><th>AFR</th><th>Global</th><th colspan="3">EUR</th><th colspan="3">AMR</th><th colspan="3">AFR</th><th colspan="3">Global</th></tr>
<tr><td>6:31827960</td><td>G/T</td><td>0.0</td><td>0.0</td><td>0.0</td><td>0.0*</td><td>TT=0</td><td>TG=0</td><td>GG=379</td><td>TT=0</td><td>TG=0</td><td>GG=170</td><td>TT=0</td><td>TG=0</td><td>GG=208</td><td>TT=0</td><td>TG=0</td><td>GG=1095</td></tr>
<tr><td>6:31829865</td><td>C/G</td><td>1.0</td><td>0.0</td><td>10.0</td><td>2.0</td><td>GG=0</td><td>GC=0</td><td>CC=379</td><td>GG=1</td><td>GC=10</td><td>CC=170</td><td>GG=0</td><td>GC=38</td><td>CC=208</td><td>GG=1</td><td>GC=48</td><td>CC=1047</td></tr>
<tr><td>6:31828365</td><td>C/T</td><td>0.0</td><td>0.0</td><td>0.2</td><td>0.05</td><td>TT=0</td><td>TC=0</td><td>CC=379</td><td>TT=0</td><td>TC=0</td><td>CC=181</td><td>TT=0</td><td>TC=1</td><td>CC=245</td><td>TT=0</td><td>TC=1</td><td>CC=1095</td></tr>
</table>

*As described in the main web page of the project, after variant identification process, two subjects had been removed from the dataset due to issues related to the global genotype analysis. Thus, variants present only in one of these two subject were retained, but indicated with zero variant allele count, as the genotype could not be surely determined. Ref, nucleotide in the reference genome sequence (hg19); alt, alternative nucleotide. MAF, Minor Allele Frequency. Populations: EUR, European; AMR, American; AFR, African.
